# What can claims data tell us about risk factors and survival of patients with hepatocellular carcinoma? Insights from a German population-based study

**DOI:** 10.3389/fonc.2025.1650982

**Published:** 2025-12-16

**Authors:** Selina Becht, Najib Ben Khaled, Simone Schrodi, Michael von Bergwelt-Baildon, Peter Buggisch, Alexander Crispin, Wolf-Peter Hofmann, Ursula Marschall, Julia Mayerle, Bernhard Mörtl, Sami Orabi, Alexander Philipp, Jörg Trojan, Tobias Weiglein, Enrico N. De Toni, Karin Berger-Thürmel

**Affiliations:** 1 Department of Medicine III, University Hospital, Ludwig-Maximilians-University of Munich, Munich, Germany; 2 Department of Medicine II, University Hospital, Ludwig-Maximilians-University of Munich, Munich, Germany; 3 Bavarian Cancer Research Center (BZKF), Munich, Germany; 4 German Cancer Consortium (DKTK), Partner Site Munich, Munich, Germany; 5 ifi-Institute for Interdisciplinary Medicine, Hamburg, Germany; 6 Association of Gastroenterologists in Private Practice, Berufsverband Niedergelassener Gastroenterologen Deutschlands, Ulm, Germany; 7 Department for Medical Information Processing, Biometry, and Epidemiology, Ludwig-Maximilians University, Munich, Germany; 8 Gastroenterologie am Bayerischen Platz, Berlin, Germany; 9 BARMER, Wuppertal, Germany; 10 Medical Clinic I, University Hospital, Goethe University Frankfurt, Frankfurt, Germany

**Keywords:** hepatocellular carcinoma, secondary data, cancer epidemiology, real-world evidence, metabolic risk factors

## Abstract

**Background:**

Geographic and temporal variations in the incidence and treatment of chronic viral hepatitis and trends in the development of metabolic and behavioral risk factors result in heterogeneous incidences of hepatocellular carcinoma (HCC). Therefore, national epidemiological information should be evaluated to identify the need for action.

**Methods:**

This retrospective observational study included adult patients with incident HCC (2016-2020). A network analysis was performed to investigate inter-relationships among risk factor diagnoses before HCC. Kaplan-Meier method and Cox proportional hazard model were used to analyze survival.

**Findings:**

A total of 2,778 patients were included. Mean age was 71.9 years (SD ±9.7); 69% were male. Most frequently documented risk factor diagnoses were diabetes mellitus (76%), obesity (56%), liver fibrosis/cirrhosis (44%), and alcohol abuse (36%). Hepatitis B and C were documented in 4% and 11% of patients. Behavioral and metabolic risk factors were 1.1-1.9-fold more frequent in men. Diabetes mellitus was the most central risk factor diagnosis co-occurring with other metabolic and behavioral risk factors. Median survival was 8.7 months.

**Interpretation:**

In this German cohort, risk factor diagnoses before HCC were multifactorial, with metabolic diseases most frequently co-occurring. Survival after HCC was poor. Controlling metabolic risk factors and surveilling at-risk populations are crucial to mitigating the incidence and improving the survival of HCC patients in Germany. Analyzing claims data enabled efficient and effective generation of epidemiological real-world evidence.

## 1 Introduction

Primary liver cancer is a highly aggressive and lethal tumor and the third common cause of cancer-related deaths worldwide, following lung and colorectal cancer (1). Hepatocellular carcinoma (HCC) comprises the majority of primary liver cancer cases (2). Most patients with HCC in Europe are diagnosed at an intermediate or advanced stage of disease, when curative treatments are no longer applicable (3). In Germany, HCC is considered a rare type of cancer, with approximately 6000 new cases reported by the German cancer registration in 2020 (4). The relative five-year and ten-year survival rates after the diagnosis of HCC in Germany are 17% and 13%, respectively (5).

HCC commonly develops as a result of a chronic liver disease such as cirrhosis. Chronic viral hepatitis is so far the primary cause of chronic liver disease and subsequent HCC (6). However, other risk factors, including chronic alcohol consumption, obesity, and metabolic diseases, also increase the likelihood of HCC (6–8). In recent years, the proportion of HCC patients without viral hepatitis has increased, and the etiologies of HCC have shifted from viral to non-viral (6–8). Particularly in developed countries, such as those in Europe and North America, successful interventions against hepatitis B virus (HBV) and hepatitis C virus (HCV) infections have been implemented (9, 10), whereas the prevalence rates of alcohol-related risk factors and metabolic diseases, such as metabolic dysfunction-associated steatotic liver disease (MASLD) and diabetes mellitus, are rapidly increasing (11). In a French cohort, the prevalence of MASLD-related HCC rose from 3% to 20% between 1995 and 2014, and the proportion of HCC caused by HCV decreased from 43% to 20% (12). Because of significant geographical and temporal variations in the prevalence of risk factors and differences in healthcare systems, the causes underlying HCC vary globally (6, 13). Regional differences have been observed in Europe as well. In socioeconomically disadvantaged regions, a higher burden of behavioral risk factors, restricted access to HBV vaccination and HCV treatment, and a subsequently higher incidence of HCC have been observed (14).

Because of the aforementioned geographical and temporal variations in the epidemiology of HCC, epidemiological knowledge of HCC among national cohorts is imperative to identify relevant risk factors and enhance targeted prevention and surveillance measures to reduce the incidence and healthcare burden of HCC. Currently, there is no contemporary real-world evidence of risk factors and survival of patients with HCC in Germany. This study aims to describe patient characteristics, the occurrence of previous risk factor diagnoses, and survival of a large population of patients with HCC in Germany based on health insurance claims data.

## 2 Methods

### 2.1 Study design and data sources

This retrospective cohort study was based on health insurance claims data from BARMER (Berlin, Germany), which is the second largest health insurance company in Germany, providing insurance coverage for approximately 8.7 million citizens in 2022. For each insured individual, the database contains anonymized longitudinal information regarding demographics (e.g., age, sex, place of residence, and date of death), outpatient care, inpatient care, drug prescriptions, therapeutic devices, and sick leave. The inpatient setting covers all hospital admissions, whereas the outpatient setting comprises ambulatory healthcare services provided by office-based physicians and hospital outpatient clinics. Health insurance claims data are primarily obtained for billing and reimbursement purposes. All diagnoses relevant to the patients’ treatment are routinely reported by physicians, hospitals, pharmacies, and other healthcare providers to the respective health insurance company. Diagnoses provided in health insurance claims data are coded according to the German Modification of the International Statistical Classification of Diseases and Related Health Problems, 10^th^ Revision (ICD-10-GM).

Data analyses and presentations followed the Strengthening the Reporting of Observational Studies in Epidemiology (STROBE) guidelines (15). Since only anonymized data from the BARMER was used, no ethics approval or consent to participate was needed. This is in accordance with national (General Data Protection Regulation) and European (§ 78 SGB) regulations, as well as German guidelines for Good Practice of Secondary Data Analysis by Swart et al. (2015) (16).

### 2.2 Patient identification

Patients with incident HCC were identified according to ICD-10-GM code C22.0 between 1 January 2016 and 31 December 2020. Patients aged ≥18 years with either one primary diagnosis of HCC in an inpatient setting or two confirmed diagnoses of HCC in an office-based or outpatient hospital setting during consecutive quarters were included. Patients with previously confirmed cancer diagnoses other than carcinoma *in situ* or non-melanoma skin cancer within 5 years prior to the HCC diagnosis were excluded because these diseases can influence the prognosis and treatment options for patients with HCC. Furthermore, these criteria distinguished incident HCC cases from prevalent or recurrent HCC cases and excluded uncertain diagnoses and liver metastases from other cancers documented as HCC. Individuals without BARMER insurance coverage for at least 10 years before and 2 years after the date of the HCC diagnosis (or unless death occurred before the diagnosis) and those insured by Deutsche BKK, a health insurance that integrated into BARMER in 2017, were excluded to ensure sufficient insurance records during the observation period. The index date of the first HCC diagnosis was defined as the date of the earliest inpatient or outpatient visit with a recorded diagnosis of HCC.

### 2.3 Study variables

#### 2.3.1 Comorbidities

Comorbidities were determined using the Charlson Comorbidity Index (CCI). The algorithm of Quan et al. (2011) and the respective ICD-10-GM codes were used to identify relevant diagnoses and calculate the CCI (17). Categories including diagnoses of malignant neoplasms were not considered in the calculation of the CCI because they could be related to or result from the index disease HCC. Comorbidities at the time of the HCC diagnosis were identified by either one documented primary or secondary diagnosis in an inpatient setting or two confirmed diagnoses in an outpatient setting during two consecutive quarters 12 months before or 3 months after the HCC diagnosis. The calculated CCI was divided into four categories (0, 1-3, 4-6, and ≥7) to describe the burden of the comorbidity and assess its impact on survival.

#### 2.3.2 Risk factors

Risk factor diagnoses prior to the HCC diagnosis were identified by retrieving either one primary or secondary diagnosis in an inpatient setting or two confirmed diagnoses in an outpatient setting during consecutive quarters within ten years before the index date. The following diagnoses were considered when assessing the occurrences of risk factors before the HCC diagnosis in this cohort: obesity, diabetes mellitus, alcohol abuse, alcohol-related liver disease, MASLD, fibrosis/cirrhosis, chronic viral hepatitis, other hepatitis, iron metabolism disorders, plasma protein metabolism disorders, unspecified inflammatory liver disease, toxic liver disease, and hepatic failure. ICD-10-GM codes were used to identify risk factor diagnoses in the health insurance claims data. MASLD, according to the new nomenclature for steatotic liver disease (18), is not yet available in the ICD-10-GM system. Hence, the diagnosis was defined based on a combination of several ICD-10-GM codes (see **Supplementary Table 1** for the respective codes).

### 2.4 Statistical analysis

Characteristics of patients were determined using descriptive statistics such as absolute numbers and percentages for categorical variables and mean with standard deviation for the continuous variable patient’s age. Based on a co-occurrence matrix, a network analysis was performed to investigate the inter-relationships among documented risk factor diagnoses. Each diagnosis was represented by a node in the network. Weights were calculated to visualize nodes dependent on the frequency of diagnoses and the edges between nodes dependent on the frequency of the co-occurrence of diagnoses (19). Overall survival was calculated from the index date of the HCC diagnosis to the date of death from any cause. Patients with no documented date of death were censored on 31 December 2022. The observed overall survival was estimated using the Kaplan–Meier method. The effects of age, sex, CCI, and HCC-associated risk factor diagnoses on survival were assessed using multivariate Cox proportional regression analysis. The forward sequential method was used as the variable selection method for the Cox regression model (20). Hazard ratios (HRs) and 95% confidence intervals (95% CIs) were presented.

A significance level of α=0.05 was prespecified for all statistical tests. Data processing and statistical analyses were conducted using SAS software (version 9.4; SAS Institute Inc., Cary, NC, USA) and R software (version 4.2.1; R Foundation, Vienna, Austria).

## 3 Results

### 3.1 Patient cohort

**Figure 1:**
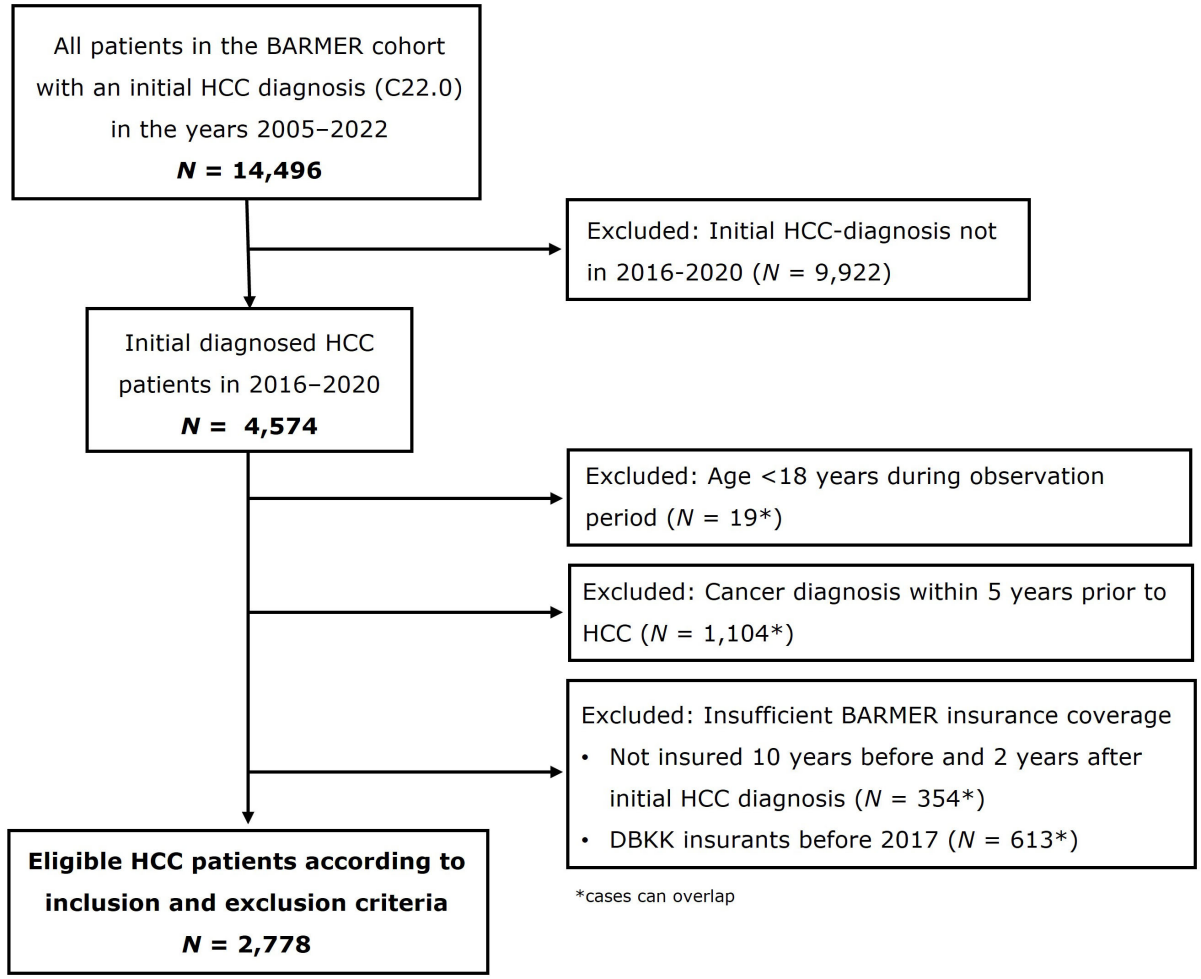
Study flowchart of patient identification. Similiar figure is already published in: Schrodi et al. (21).

A progressive approach was used to identify eligible patients with an incident HCC diagnosis (**Figure 1**). Between 1 January 2016 and 31 December 2020, 4,574 patients with an incident HCC diagnosis were identified. After applying the inclusion and exclusion criteria, 2,778 patients were included in the subsequent analyses.

### 3.2 Patient characteristics

Most patients (69.4%) in the cohort were male. The mean age of patients at the time of the HCC diagnosis was 71.9 years (standard deviation, ± 9.7; range, 30–97 years). Regarding comorbidities, the majority of the cohort (62.0%) had a CCI ≥4 at the time of the HCC diagnosis. No comorbidity burden was recorded for 6.5% of the patients (**Table 1**). Mild liver disease (40.3%) and moderate/severe liver disease (37.5%) were the most frequently documented comorbid conditions, followed by renal disease (34.4%), diabetes mellitus with chronic complications (33.4%), and congestive heart failure (30.7%) (**Supplementary Table 2**).

**Table 1:** Baseline characteristics of HCC patients at the time of initial diagnosis over the period 2016-2020 (*N* = 2,778).

Characteristics	HCC patients *n* (%)
Total	2778 (100.0)
Mean age [SD]	71.9 [± 9.7]
Age group (%)	
≤49	46 (1.7)
50-59	254 (9.1)
60-69	798 (28.7)
70-79	1042 (37.5)
≥80	638 (23.0)
Sex (%)	
Female	850 (30.6)
Male	1928 (69.4)
Charlson Comorbidity Index (%)	
0	180 (6.5)
1-3	875 (31.5)
4-6	1267 (45.6)
≥7	455 (16.4)

HCC, hepatocellular carcinoma; SD, Standard deviation.

### 3.3 Distribution of risk factor diagnoses and network analysis

**Figure 2:**
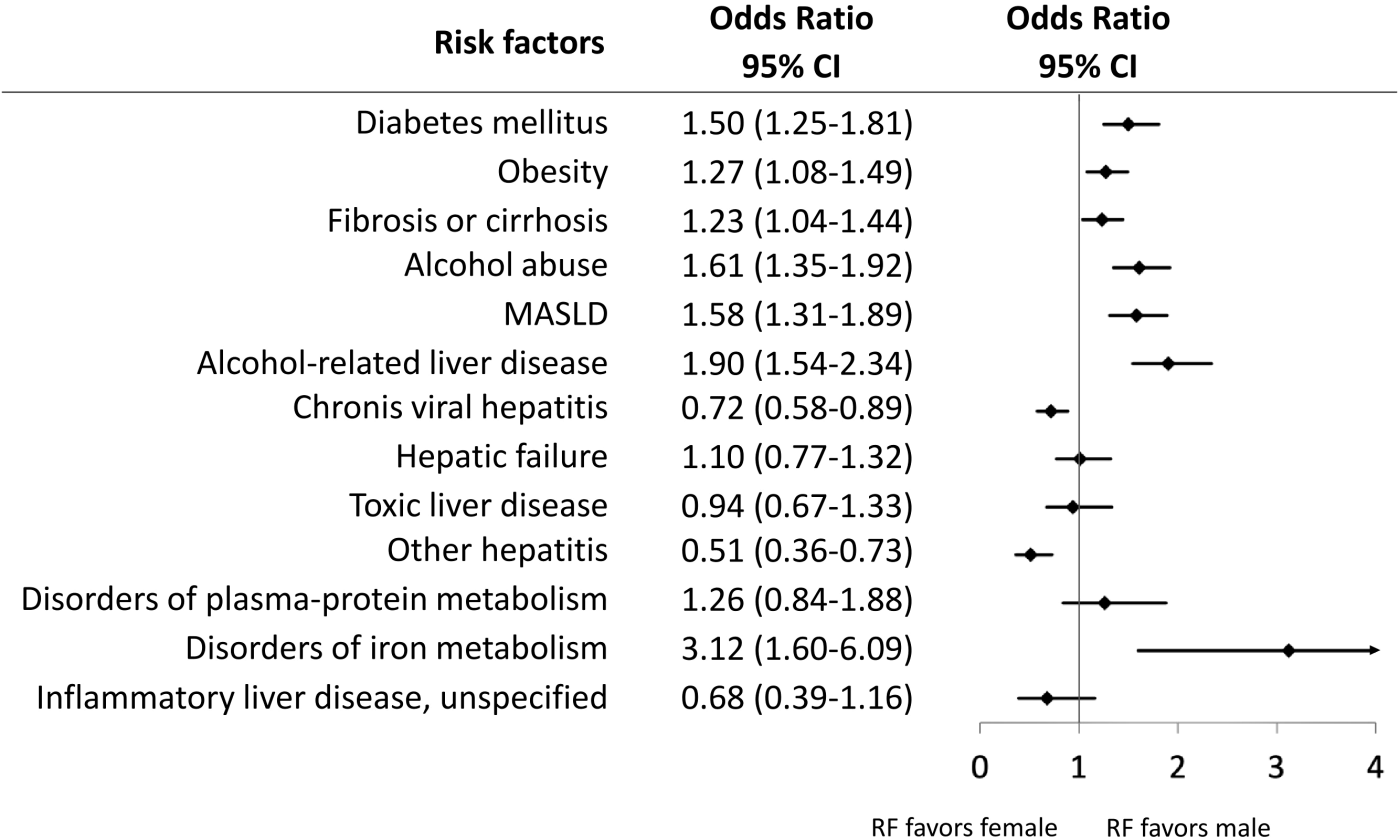
Forest plots of the female to male odds ratios (OR) and 95% confidence intervals (CI) for HCC risk factors (RF).

**Figure 3:**
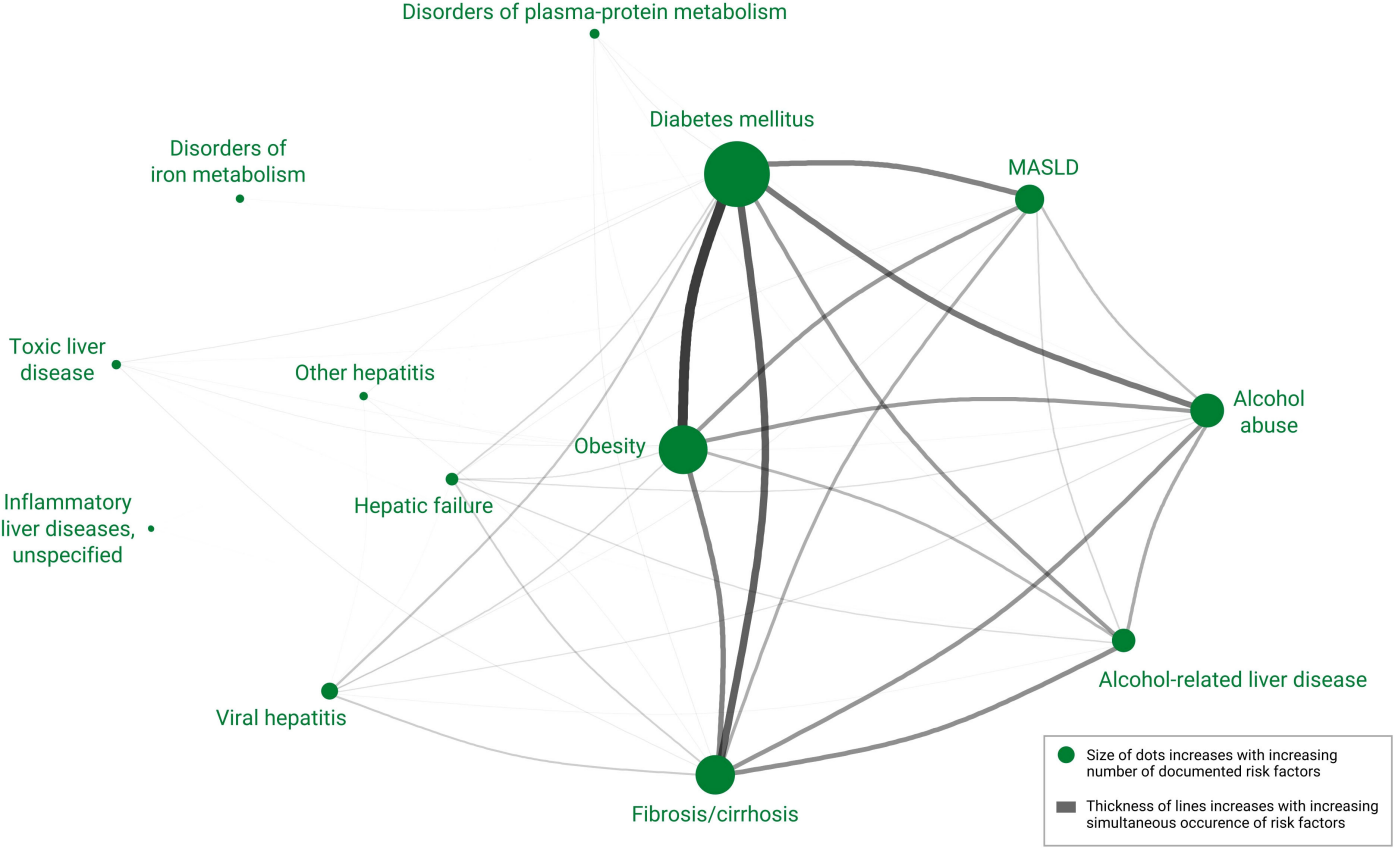
Network showing associations between different risk factors in patients with HCC diagnosis. Size of nodes is based on the prevalence of the risk factor in the HCC cohort, and the thickness of lines is based on the number of simultaneous occurrences of the risk factors.

The proportions of risk factor diagnoses within 10 years before the HCC diagnosis are shown in **Table 2**. The five most frequently documented diagnoses were diabetes mellitus (76.3%), obesity (55.5%), liver fibrosis/cirrhosis (43.8%), alcohol abuse (36.3%), and MASLD (30.8%). HBV and HCV were documented for 4.3% and 10.9% of the patients, respectively. Significant differences in the risk factor diagnoses were observed between men and women (**Figure 2**). The odds of behavioral and metabolic risk factors of male patients were 1.1- to 1.9-fold higher than those of female patients with HCC. As shown in **Figure 3**, the network of diseases documented within 10 years preceding the HCC diagnosis revealed the multifactorial nature of the disease. The network comprised 13 nodes representing each determined diagnosis and 13,506 edges representing the co-occurrence of two risk factor diagnoses. The most central node in the network was diabetes mellitus, which most frequently co-occurred with obesity, followed by liver fibrosis/cirrhosis, alcohol abuse, alcohol-related liver disease, and MASLD.

**Table 2:** HCC risk factors recorded within 10 years prior HCC diagnosis – several possible risk factors in the same patient (*N* = 2,778).

HCC risk factors	*n* (%)
Diabetes mellitus	2119 (76.3)
Obesity	1543 (55.5)
Fibrosis and cirrhosis	1216 (43.8)
Alcohol abuse	1009 (36.3)
MASLD	840 (30.8)
Alcohol-related liver disease	656 (23.6)
Chronic viral hepatitis	423 (15.2)
Hepatic failure	276 (9.9)
Toxic liver disease	157 (5.7)
Other hepatitis	130 (4.7)
Disorders of plasma-protein metabolism	130 (4.7)
Disorders of iron metabolism	79 (2.8)
Inflammatory liver disease, unspecified	56 (2.0)

HCC, hepatocellular carcinoma; MASLD, metabolic dysfunction-associated steatotic liver disease.

### 3.4 Survival

**Figure 4:**
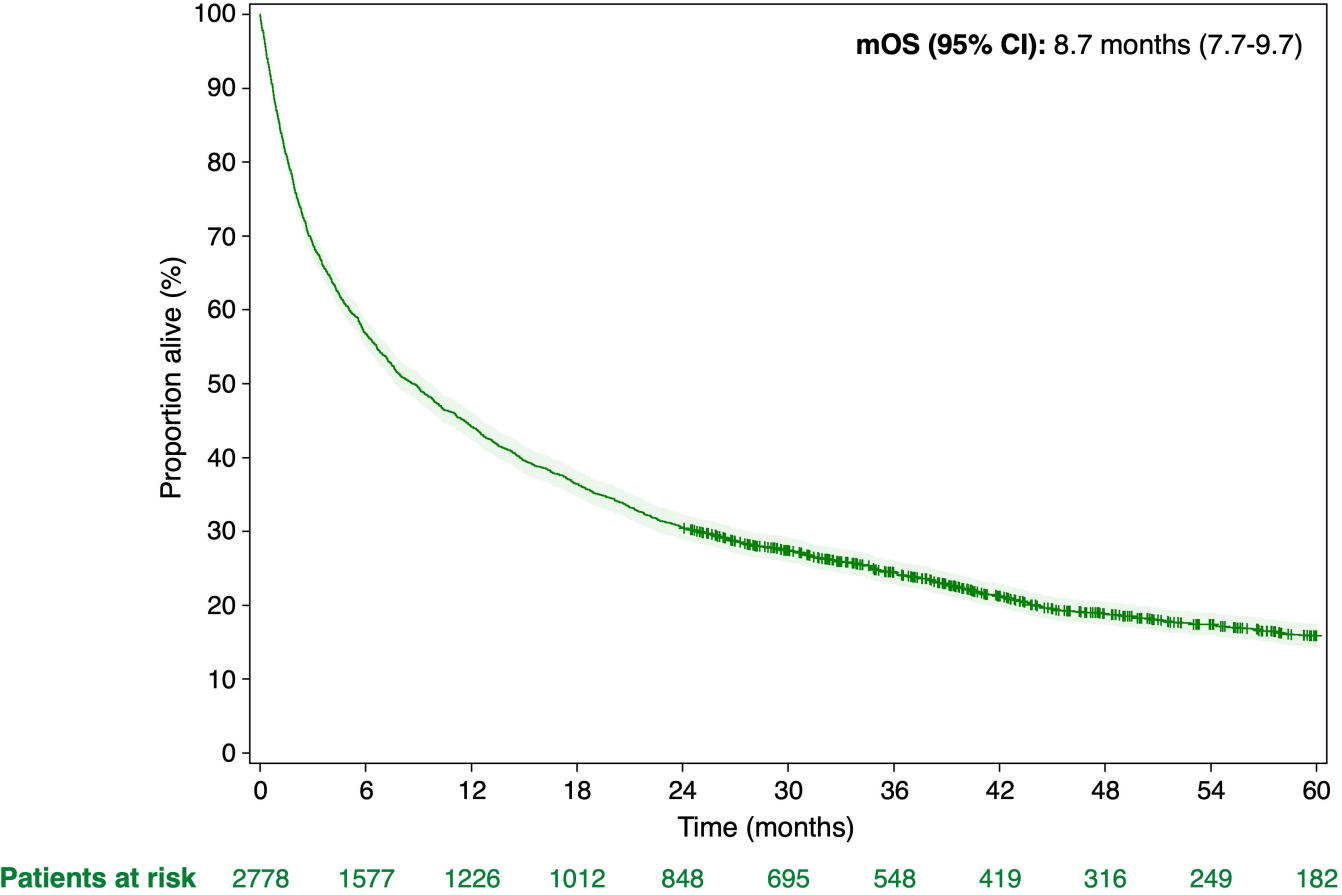
Analysis of overall survival (OS) according to the Kaplan-Meier method in patients with HCC.

The median overall survival after the HCC diagnosis was 8.7 months (95% CI, 7.7-9.7 months). After 1 year and 5 years, the estimated survival rates were 44% and 16%, respectively (**Figure 4**).

The multivariate Cox regression analysis revealed that age, CCI, chronic viral hepatitis, MASLD, and alcohol abuse were independent predictors of patients’ survival (**Table 3**). The median survival time following HCC diagnosis ranged from 52.2 months in patients younger than 50 years to 5.6 months in those aged 80 years and older (HR, 3.25; 95% CI, 2.13-4.94; p<0.0001). A higher CCI was associated with reduced survival time Patients with no or a low burden of comorbidities had prolonged survival (p<0.0001). Regarding risk factor diagnosis, patients with documented alcohol abuse had reduced survival compared to patients without alcohol abuse prior to the HCC diagnosis (HR, 1.15; 95% CI, 1.05-1.25; p=0.0021). Patients diagnosed with chronic viral hepatitis (HR, 0.81; 95% CI, 0.72-0.91; p=0.0005) or MASLD (HR, 0.85; 95% CI, 0.77-0.93; p=0.0004) had prolonged survival. The other investigated risk factors were not identified as independent predictors of survival.

**Table 3:** Stratified median survival and multivariate Cox proportional regression analysis of the survival after HCC diagnosis (*N* = 2,778).

Characteristics	Median survival (months)	*Multivariate analysis*	
		Hazard Ratio [95% CI]	*P*-value
Age group			<0.0001
≤49	52.2	*ref.*	
50-59	14.5	1.88 [1.22-2.92]	
60-69	12.0	2.10 [1.38-3.19]	
70-79	8.1	2.49 [1.64-3.78]	
≥80	5.6	3.25 [2.13-4.94]	
Charlson Comorbidity Index			<0.0001
0	13.2	*ref.*	
1-3	13.5	1.05 [0.87-1.27]	
4-6	7.8	1.33 [1.11-1.60]	
≥7	3.7	1.87 [1.53-2.28]	
Chronic viral hepatitis (no as ref.)	12.6	0.81 [0.72-0.91]	0.0005
MASLD (no as ref.)	11.3	0.85 [0.77-0.93]	0.0004
Alcohol abuse (no as ref.)	7.7	1.15 [1.05-1.25]	0.0021

HCC, hepatocellular carcinoma; CCI, Charlson Comorbidity Index; MASLD, metabolic dysfunction-associated steatotic liver disease; CI, confidence interval.

## 4 Discussion

This retrospective observational study provides contemporary insights regarding risk factor diagnoses and survival of 2,778 patients with HCC based on claims data from a large German health insurance company. To the best of our knowledge, this is the first extensive epidemiological analysis of patients with HCC in Germany. Chronic HBV or HCV was documented for 15% of the included patients before the HCC diagnosis. Metabolic diseases and behavioral risk factor diagnoses affected the majority of patients. The network analysis highlighted the relevance and inter-relationships of these non-viral risk diseases of patients with HCC. Survival was highly dependent on age and CCI at the time of diagnosis. Younger patients and those with a low comorbidity burden had significantly prolonged survival.

### 4.1 Distributions of age and sex

The mean age of patients in this study cohort was 71.9 years. Patients between 70 and 79 years comprised the highest proportion of the cohort (38%). The age distribution was comparable to that of other German HCC cohorts based on population-level cancer registry data (mean age, 68.5 years) (22) and health insurance claims data (mean age, 68.9-71.9 years) (23, 24). In Germany, the proportion of inhabitants between 70 and 79 years of age is projected to increase from 9% to 13% between 2023 and 2040 (25). Considering the predicted demographic changes, a substantial increase in the number of patients with HCC is expected.

Approximately one-third of patients with incident HCC were female. However, previous epidemiological studies have demonstrated that the incidence of HCC for men is up to five-fold higher than that for women (2). This deviation may be explained by the structure of the BARMER, which includes proportionally more women compared to the general population in Germany.

### 4.2 Prevalence of risk factor diagnoses prior to the HCC diagnosis

In this German cohort, only 15% of the patients were affected by chronic viral hepatitis, whereas metabolic diseases were the most frequently documented diseases prior to HCC. The network analysis identified the multifactorial nature of risk factor diagnoses prior to HCC and highlighted the less prominent role of viral infections compared to metabolic diseases. These findings are consistent with previously published population-based studies in Europe (14, 26) and the United States (27). The observed inferior role of chronic viral hepatitis in patients with HCC aligned with the recently reported shift from viral to non-viral HCC (6, 13), presumably because of successful interventions against HBV and HCV and an increasing prevalence of metabolic risk factors. A retrospective analysis in France found that HCC cases caused by MASLD increased from 3% to 20% between 1995 and 2014. Simultaneously, the proportion of patients with HCC and HCV declined from 43% to 20% (12).

The odds of having behavioral and metabolic risk factors were higher for male patients than for female patients. Similar sex disparities in the epidemiological distribution of risk factors for HCC have been previously reported (28). Because of the lower proportion of male patients, as expected in population-based HCC cohorts, behavioral and metabolic risk factor diagnoses in our total cohort may have been underestimated.

In the Netherlands, approximately 20% of HCC cases develop in non-cirrhotic livers, predominantly in patients with MASLD (29). A similar proportion of patients with non-cirrhotic livers were identified in a large single-center HCC cohort in Germany (30). Contrary to these findings, liver fibrosis or cirrhosis was documented before the HCC diagnosis for only 43% of patients in our study. This comparatively low number could be attributable to the potential underestimation of affected patients with liver fibrosis or cirrhosis never seek clinical care and, therefore, remain undiagnosed (31). Because health insurance claims data are used for reimbursement purposes, diseases that were not diagnosed by a physician were not documented in the data source.

### 4.3 Survival and risk factors

In this claims data study, the observed median survival after the HCC diagnosis for all patients was 8.7 months. Previous studies in Germany using population-based cancer registry data have found slightly better survival for patients with HCC (median, 1 year) (5, 22). As expected, the median observed survival in the current study decreased significantly with increasing age decade and comorbidity index.

Additional independent predictors for the survival of patients with HCC that were identified were chronic viral hepatitis, MASLD, and alcohol abuse. Consistent with a previous retrospective study based on the SEER-Medicare database (32) and a prospective study in France (33), we found that alcohol abuse leads to poorer prognoses for patients with HCC, whereas chronic viral hepatitis is associated with prolonged survival after the diagnosis of HCC. More than half of patients with alcohol-related HCC (55%) have been diagnosed at an advanced stage of disease, whereas patients with chronic viral hepatitis are often diagnosed at an early tumor stage (30). Late-stage diagnoses and poor survival of patients with alcohol-related HCC could be explained by a lack of screening measures (34) and reduced likelihood of receiving curative treatment (35) compared to individuals with other HCC etiologies. We also found patients diagnosed with MASLD prior to HCC diagnosis had prolonged survival as compared to patients not diagnosed with one of these risk factors. Carrying a diagnosis such as viral hepatitis or MASLD may lead to being more likely to be diagnosed with HCC in earlier stages because they may be more likely to seek clinical care and receive surveillance measures (36).

Although treatment modalities were not considered in the present analysis, therapy also has a major impact on survival. In a recently published study based on the same patient cohort, we showed that patients who received curative therapy had a markedly better median survival (40.4 months) compared to those receiving non-curative treatment (9.7 months) (21).

### 4.4 The value of analyzing claims data

Almost 2,800 patients with HCC were identified for the analyses even though this type of cancer is rare in Germany (4), thus highlighting the notable advantage of analyzing health insurance claims data. This source of data comprises complete data coverage of different sectors of healthcare in Germany, including inpatient and outpatient diagnostics and treatments, resource consumption, costs, and survival by all patients in real-life conditions, independent of a predefined study purpose.

Population-based studies have revealed the varying prevalence of risk factors and subsequent HCC incidence between countries and regions. Recently, the shift from viral to non-viral causes of HCC has been observed in Europe (6, 13). A meta-analysis by Riazi et al. (2022) observed a globally increasing prevalence of MASLD in recent decades, reaching a prevalence of 37.8% in the years 2016–2019 (37). Given the rising number of patients affected by metabolic diseases such as diabetes mellitus and MASLD, particularly in countries with a high socio-demographic, the number of HCC cases caused by metabolic diseases is expected to increase further (11). Furthermore, patients with metabolic diseases are from a generation comprising individuals with mostly normal weights during childhood. Currently, approximately one of every five adolescents (21%) in Europe is overweight or obese, resulting in an increased incidence of metabolic disease burden during adulthood (38). Regarding chronic viral hepatitis, a burden of HBV and HCV infections was still reported, although Germany being considered to be a low-prevalence country (39). The HBV vaccination has been administered to between only 66% and 91% of all children in Germany; however, the goal of the World Health Organization is 95% (40). A significant proportion of patients with HCV still need to be reached by treatment programs (41). Furthermore, the large-scale movement of individuals from high-prevalence nations to European countries highlights the importance of testing risk groups, treating chronic viral hepatitis, and performing surveillance measures, which are crucial to preventing the development of late stage HCC caused by viral infections (42). Health insurance claims data provide access to contemporary data at the national level and enable analyses of the current epidemiology and regional and temporal trends.

### 4.5 Limitations

Despite the strengths and value described, this study had some limitations, which were mainly related to the nature of claims data from German health insurances. The database consisted of data from one health insurance company that provided coverage for approximately 10% of the German population. Hence, the generalizability of the study results to other populations of patients with HCC in Germany may be limited. Because of the billing and reimbursement purposes of German statutory health insurance data, recorded diagnoses are dependent on the coding quality and relevance of the reimbursement of healthcare costs, thus leading to potential bias or, from a clinical perspective, incomplete documentation. This may lead to underrepresentation or overrepresentation of the determined comorbidities or risk factors. However, the risk of overestimating diagnoses was minimized through our approach for identifying HCC cases, risk factors, and comorbidities. Furthermore, health insurance data do not capture information regarding tumor stage, histology, Eastern Cooperative Oncology Group performance status, or laboratory results for considering the liver function using the Child-Pugh score. This lack of detailed clinical information limited the ability to control relevant characteristics, such as tumor stage, that might affect the survival of patients with HCC.

### 4.6 Conclusion

In conclusion, the results of our analysis based on data from a large comprehensive German claims database revealed the relevance of the multifactorial nature of risk factors in patients with HCC. Most patients were affected by metabolic diseases, whereas only a few were affected by chronic viral hepatitis infections prior to the diagnosis of HCC. Awareness among healthcare professionals, at-risk groups, policymakers, and the general population regarding the relevance of controlling behavioral risk factors and subsequent metabolic diseases is crucial to mitigating the incidence and burden of HCC. This study demonstrated the potential of claims data sources to allow rapid collection of longitudinal data of large populations to answer pivotal clinical and political questions during healthcare research. The methodological approach used during this study can serve as a template for closing epidemiological information gaps using health insurance claims data. Based on our work, subsequent research should aim to identify trends in risk factor incidences and evaluate their long-term impact on HCC incidence and mortality. Therefore, studies linking health insurance claims data with German cancer registry data are needed to enable robust survival analyses by incorporating information on tumor stage and performance status. Furthermore, including complementary information regarding diagnostics, treatment patterns, and costs in future analyses is essential to provide a comprehensive view of prevention and surveillance measures, outcomes, and health economic consequences of HCC.

## Data Availability

The data analyzed in this study is subject to the following licenses/restrictions: The anonymized data underlying this study belong to BARMER health insurance. The data are not publicly available due to protection of data privacy of the insured individuals by the BARMER. Requests to access these datasets should be directed to UM, ursula.marschall@barmer.de.
