# Supplementary material for: What can claims data tell us about risk factors and survival of patients with hepatocellular carcinoma? Insights from a German population-based study
Source: Front Oncol. 2025 Dec 16;15:1650982. doi: 10.3389/fonc.2025.1650982 (PMC12750458; doi:10.3389/fonc.2025.1650982)
Supplement: Supplementary file 1 [file Table1.docx]

Supplementary Material

**Supplementary Table 1. List of ICD-10-GM codes used for the identification of risk factor diagnoses in patients with hepatocellular carcinoma**

| Diagnosis | ICD-10-GM Codes |
| --- | --- |
| Diabetes mellitus | E10-14 |
| Obesity | E65-66 |
| Fibrosis/cirrhosis | K70.3, K74, K71.7 |
| Alcohol abuse | F10.1-10.9, G62.1, K29.2 |
| Alcohol-related liver disease | K70.0, K70.1, K70.2, K70.3 |
| MASLD | K76.0, K75.8 AND (E10-14 OR E65-66 OR I10-13, I15 OR E78.0-5) |
| Chronic viral hepatitis | B18, B19, Z22.5 |
| Hepatic failure | K72 |
| Toxic liver disease | K71 |
| Other hepatitis | K73, K75.2-4 |
| Disorders of plasma-protein metabolism | E88.0 |
| Disorders of iron metabolism | E83.1 |
| Inflammatory liver disease, unspecified | K75.8, K75.9 |
| MASLD, Metabolic dysfunction-associated steatotic liver disease; ICD-10-GM, International Classification of Diseases version 10 German | |

**Supplementary Table 2. Comorbidity prevalence in absolute numbers and percentage of patients with hepatocellular carcinoma (N = 2,778) at the time of initial diagnosis over the period 2016-2020**

| **Comorbidity** | **n (%)** |
| --- | --- |
| Myocardial infarction | 243 (8.8) |
| Congestive heart failure | 854 (30.7) |
| Peripheral vascular disease | 840 (30.2) |
| Cerebrovascular disease | 604 (21.7) |
| Dementia | 326 (8.5) |
| Chronic pulmonary disease | 789 (28.4) |
| Rheumatic disease | 111 (4.0) |
| Peptic ulcer disease | 330 (11.9) |
| Diabetes without chronic complication | 565 (20.3) |
| Diabetes with chronic complication | 928 (33.4) |
| Hemiplegia or paraplegia | 148 (5.3) |
| Renal disease | 955 (34.4) |
| Mild liver disease | 1119 (40.3) |
| Moderate or severe liver disease | 1043 (37.5) |
| AIDS/HIV | 4 (0.1) |
